# SipA Activation of Caspase-3 Is a Decisive Mediator of Host Cell Survival at Early Stages of Salmonella enterica Serovar Typhimurium Infection

**DOI:** 10.1128/IAI.00393-17

**Published:** 2017-08-18

**Authors:** Anne McIntosh, Lynsey M. Meikle, Michael J. Ormsby, Beth A. McCormick, John M. Christie, James M. Brewer, Mark Roberts, Daniel M. Wall

**Affiliations:** a Institute of Infection, Immunity and Inflammation, College of Medical, Veterinary and Life Sciences, University of Glasgow, Glasgow, United Kingdom; b Department of Microbiology and Physiological Systems, University of Massachusetts Medical School, Worcester, Massachusetts, USA; c Institute of Molecular Cell and Systems Biology, University of Glasgow, Glasgow, United Kingdom; d Institute of Comparative Medicine, Faculty of Veterinary Medicine, University of Glasgow, Glasgow, United Kingdom; University of California, Davis

**Keywords:** Salmonella, immune cells, imaging, SipA, caspase-3, caspases, host-pathogen interactions, microscopy

## Abstract

Salmonella invasion protein A (SipA) is a dual-function effector protein that plays roles in both actin polymerization and caspase-3 activation in intestinal epithelial cells. To date its function in other cell types has remained largely unknown despite its expression in multiple cell types and its extracellular secretion during infection. Here we show that in macrophages SipA induces increased caspase-3 activation early in infection. This activation required a threshold level of SipA linked to multiplicity of infection and may be a limiting factor controlling bacterial numbers in infected macrophages. In polymorphonuclear leukocytes, SipA or other Salmonella pathogenicity island 1 effectors had no effect on induction of caspase-3 activation either alone or in the presence of whole bacteria. Tagging of SipA with the small fluorescent phiLOV tag, which can pass through the type three secretion system, allowed visualization and quantification of caspase-3 activation by SipA-phiLOV in macrophages. Additionally, SipA-phiLOV activation of caspase-3 could be tracked in the intestine through multiphoton laser scanning microscopy in an *ex vivo* intestinal model. This allowed visualization of areas where the intestinal epithelium had been compromised and demonstrated the potential use of this fluorescent tag for *in vivo* tracking of individual effectors.

## INTRODUCTION

Salmonella enterica serovar Typhimurium is a Gram-negative bacterial pathogen that causes a self-limiting gastroenteritis with rare complications in the immunocompromised. Infection by *S*. Typhimurium is mediated by an array of effector proteins that are delivered sequentially and temporally into targeted host cells through a needle-like apparatus, the type three secretion system (T3SS) (1). These effectors then hijack the structural, immunological, and life cycle processes of the host cell (2).

While these effector proteins have been characterized and assigned to pathogenicity islands, the best described being Salmonella pathogenicity island 1 (SPI-1) and SPI-2, the regulation of their expression within distinct cell types or even outside host cells is not clear-cut. Up to 90% of SPI-1 effectors are released extracellularly by *S*. Typhimurium during infection, indicating that the process of effector release and translocation into host cells is at best wasteful, given our current understanding (3). While effectors such as Salmonella invasion protein A (SipA) and Salmonella invasion protein C (SipC) have defined functions in driving actin polymerization during invasion of intestinal epithelial cells, these same effectors have, according to our current knowledge, little role to play in circulating immune cells where bacteria are actively phagocytosed and actin polymerization is not driven by the pathogen (4–6). Therefore, while the effectors encoded on SPI-1 and SPI-2 play roles in initial invasion and persistence, respectively, some of these same effectors from SPI-1, such as SipA, are also expressed during the more persistent phase of infection (7–9).

The first effector protein delivered into host intestinal epithelial cells after initiation of infection is SipA. This effector plays a crucial role in invasion, promoting actin polymerization that leads to membrane ruffling and bacterial uptake into the intestinal epithelium (5). We previously identified a second role for this effector in inducing activation of the crucial host apoptotic mediator, the enzyme caspase-3 (10). This led to SipA being subsequently processed by caspase-3 into two functional domains, with the C-terminal domain free to polymerize actin while the N-terminal domain induced polymorphonuclear leukocyte (PMN) migration through the induction of eicosanoid release by the intestinal epithelium (11).

While SipA therefore plays well defined roles in invasion of the intestinal epithelium and the associated inflammatory response, its role in other cell types where it is also expressed during infection remain largely unclear. Given that after crossing the epithelium *S*. Typhimurium can penetrate deeper into tissues and potentially cause systemic infection after uptake by circulating immune cells such as macrophages and dendritic cells, the roles of apparently redundant effectors such as SipA that are expressed within these cells need to be better understood (12, 13). Within these cells *S*. Typhimurium can survive by establishing a replicative niche called the Salmonella-containing vacuole (SCV) through the use of effector proteins from SPI-2 (14, 15). However, SipA is also expressed within macrophages, and as phagocytosis of *S*. Typhimurium is a passive process independent of actin polymerization, this leads to the questions of what role this SPI-1 effector plays within these cells and if this role may be mediated by or directed at caspase-3 (8, 9, 16, 17).

As previously stated, secretion of effector proteins during *S*. Typhimurium infection is an imprecise process with extracellular secretion due to the fact that secretion and translocation of the effectors are not intrinsically linked (3). As *S*. Typhimurium induces a rapid inflammatory response in the form of PMN transmigration of the intestine, we speculated that SipA may be influencing PMNs in the intestinal lumen or at the epithelial surface due to its ability to induce caspase-3 activation (10). Previous work has highlighted the role of apoptosis of PMNs in infection, with apoptosis of PMNs either conversely contributing to resolution of infection, in the case of pathogens such as Burkholderia cepacia, Borrelia hermsii, Listeria monocytogenes, Fusobacterium nucleatum, and Staphylococcus aureus, or contributing to the infection process and exacerbating infection, in the case of Streptococcus pyogenes (18). In the case of F. nucleatum, the mediator of this effect was suspected to be proteinaceous in nature and extracellular. Therefore, we speculated that SipA, or another SPI-1 effector, may play a similar role in influencing the PMN life span due to its expression and release when the bacteria are in contact with PMNs at the apical surface of the epithelium (10, 11).

In order to study the role of SipA in greater detail *in vitro* and *ex vivo*, we employed a C-terminal phiLOV tag (19). This small plant-derived tag can fluoresce under anaerobic conditions, recovers well after photobleaching, and has shown promise for the tracking of single effector proteins that are delivered through the Escherichia coli T3SS (20–22). The phiLOV tag overcomes some of the limitations of other fluorescent tags that are either too large or dependent on binding to other proteins to induce their fluorescence upon entry into the target host cell (23, 24).

Here we show that a single effector protein, SipA, plays complementary roles in macrophages *in vitro* and in intestinal epithelial cells *ex vivo* in promoting infection through its early induction of caspase-3 activity. In macrophages, we speculate that induction of caspase-3 activity leading to apoptosis in response to SipA levels allows control of intracellular bacterial numbers, ensuring a wide distribution of low numbers of initially infecting bacteria. Surprisingly, and despite their release extracellularly in the vicinity of PMNs in the intestine, neither SipA nor other effectors had any discernible effect on apoptosis or necrosis in PMNs, in contrast to the case for other pathogens. Through the use of the phiLOV tag, we tracked caspase-3 activation in macrophages infected by SipA-phiLOV-expressing *S*. Typhimurium. SipA-phiLOV was also seen to induce caspase-3 activation in ileal loops visualized *ex vivo* using multiphoton laser scanning microscopy (MPLSM). This is the first time an effector protein in isolation has been visualized activating a distinct pathway in the intestine in this manner, and this provides a new means to study the role of these bacterial proteins *in vitro* and potentially *in vivo*.

## RESULTS

### SipA plays a role in regulating host caspase-3 expression in RAW264.7 macrophages but not PMNs.

**FIG 1:**
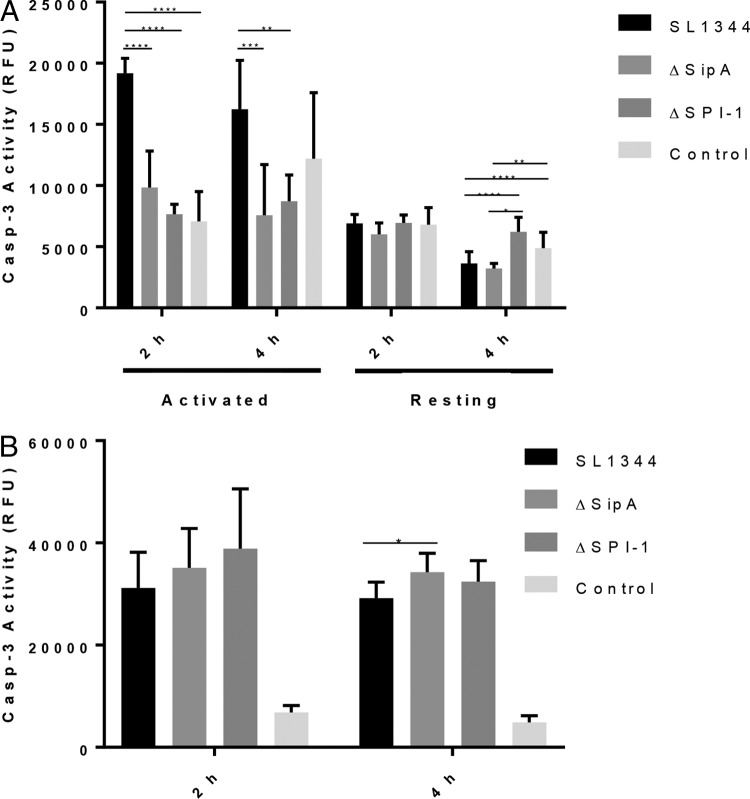
Activation of caspase-3 in RAW264.7 macrophages by SipA is dependent on activation state and MOI. (A) SipA induces increased caspase-3 activation in infected activated macrophages over the first 4 h of infection at an MOI of 10. This increase in caspase-3 activation is dependent on the activation state of RAW264.7 cells, with resting cells not exhibiting a SipA-dependent increase in activity. (B) An MOI of 100 also removes the SipA dependence of increased caspase-3 activity in activated macrophages. The data presented are representative of three independent infections, and statistically significant differences are denoted. *P* values: *, <0.05; **, <0.01; ***, <0.001; ****, <0.0001.

Increased caspase-3 activation occurs in SL1344-infected intestinal epithelial cells over the first 5 h of infection, with the effector protein SipA playing a central role (10). Here we have investigated whether SipA is capable of inducing a similar response in immune cells, specifically macrophages. Caspase-3 activity was measured in RAW264.7 murine macrophages infected with wild-type SL1344, a ΔSipA mutant, or a ΔSPI-1 mutant at 2 and 4 h postinfection (hpi). Caspase-3 activation was decreased in both mutants at 2 and 4 hpi, indicating that activation of caspase-3 in infected RAW264.7 macrophages is dependent on SPI-1 and, in particular, SipA (Fig. 1A). This decrease was independent of the bacterial load within cells, which was not significantly different across all infected cells (see Fig. S1A in the supplemental material). Notably, increased caspase-3 activity in response to SipA was dependent on the activation state of the RAW264.7 cells and was not seen in resting macrophages (Fig. 1A). The role of SipA in induction of caspase-3 activity was also directly related to the multiplicity of infection (MOI). When the MOI was increased 10-fold to 100, the dependence on SipA disappeared (Fig. 1B). To establish that any increase in cell death occurring through apoptosis was independent of necrosis, cytotoxicity was assayed by measuring lactate dehydrogenase (LDH) release postinfection. Since there was no significant difference in the levels of LDH released by macrophages infected with either the wild-type or mutant strains, the effect of SipA on macrophages was deemed to be independent of necrosis (Fig. S1B).

**FIG 2:**
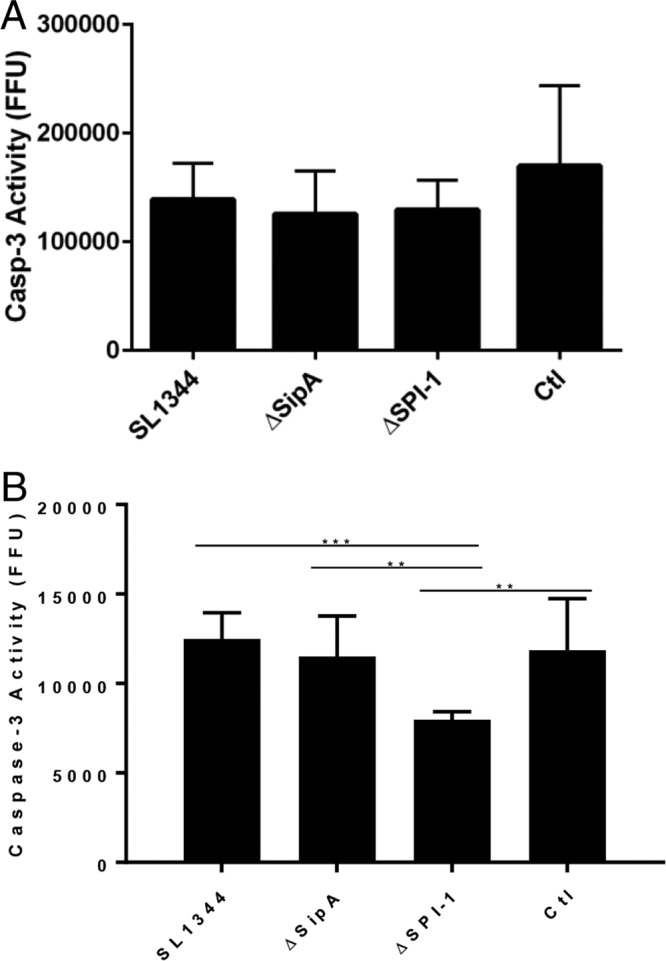
Secreted effectors play no role in protecting *S*. Typhimurium from PMNs. (A) Whole bacteria were added to PMN cultures and incubated for 6 h, but no increase in caspase-3 activity was observed in PMNs upon addition of the different strains. (B) Bacterial supernatants cleared of bacteria by filtration and equilibrated to ensure identical protein content were added to PMN cultures and left for 6 h. The SL1344 strain expressing SipA showed no increased induction of caspase-3 activation compared to the ΔSipA strain. However, an unexplained statistically significant and reproducible reduction in caspase-3 levels in ΔSPI-1-treated PMNs was observed. The data presented are representative of three independent experiments, and statistically significant differences are denoted (**, *P* < 0.01; ***, *P* < 0.001).

As a potential proteinaceous mediator of PMN apoptosis had previously been described in the literature as being released by pathogenic bacteria during infection, we next examined the potential roles of SipA and SPI-1 in the induction of caspase-3 activity in PMNs (18). We envisaged a similar role for the SPI-1 effector SipA as an anti-immune cell factor that protects *S*. Typhimurium from PMNs arriving in high numbers to sites of infection in the intestine (11). PMNs isolated from human blood were treated with either whole bacteria or filtered supernatants containing effector proteins but no bacteria. Wild-type, ΔSPI-1, or ΔSipA bacteria were insufficient to induce any changes in caspase-3 activation when added to PMN cultures (Fig. 2A). When filtered supernatants from these cultures were added directly to the cells, no significant change in caspase-3 activity was noted in the ΔSipA mutant, while an unexplained antiapoptotic effect was noted upon addition of the ΔSPI-1 mutant strain supernatant, which lacks any SPI-1 effectors (Fig. 2B). This decrease was independent of SipA. However, as the ΔSPI-1 strain lacks any effectors and the phenomenon was independent of SipA, this was not investigated further.

### Generation of a SipA-phiLOV expression system in a ΔSipA strain.

In order to gain a better understanding of SipA induction of caspase-3 activation in host macrophages and epithelial cells, we generated fluorescently tagged SipA. SipA was cloned into a pUC57 vector (pT7) bearing a phiLOV tag at the C terminus, under the control of an IPTG (isopropyl-β-d-thiogalactopyranoside)-inducible promoter, to generate pSipA-phiLOV. phiLOV is a derivative of the light, oxygen, and voltage (LOV)-sensing flavoprotein domain from plant phototropins and has many attributes that lend it to imaging (25). It fluoresces in a similar part of the spectrum as green fluorescent protein (GFP), but it is significantly smaller than GFP (13 kDa versus 27 kDa), which permits secretion of tagged proteins through the T3SS (19, 20). At 13 kDa it is also less likely to interfere with protein folding, and in addition, it is not dependent on oxygen for activity, making it suitable for anaerobic environments. The pSipA-phiLOV construct was introduced into the *S*. Typhimurium ΔSipA strain to generate a complemented SipA-tagged strain (ΔSipA/pSipA-phiLOV).

**FIG 3:**
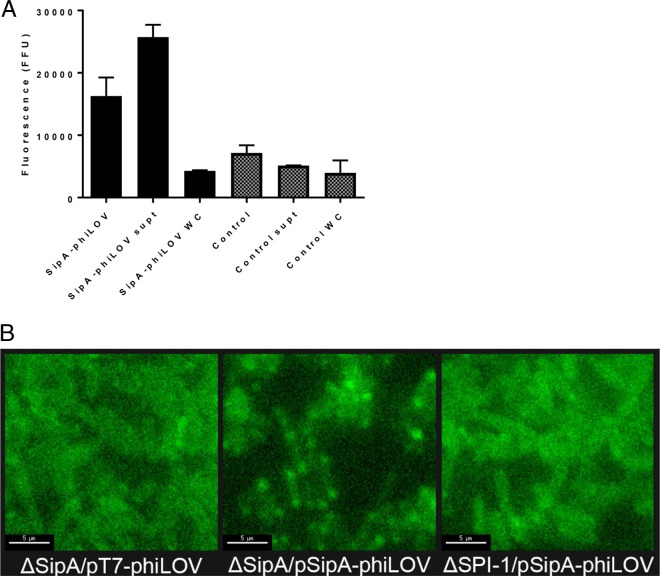
SipA-phiLOV is expressed by ΔSipA *S*. Typhimurium and is secreted through the T3SS. (A) ΔSipA *S*. Typhimurium expressing SipA-phiLOV exhibited increased fluorescence upon growth under inducing conditions. This fluorescence was primarily in the supernatant, as phiLOV is known to be secreted through the T3SS. (B) SipA-phiLOV was detected at polar focal points within expressing cells, and these focal points were speculated to be at points where T3SSs were present in anticipation of being secreted. These foci of fluorescence were absent in strains lacking SipA (ΔSipA/pT7-phiLOV) and lacking a T3SS (ΔSPI-1/pSipA-phiLOV). Scale bar, 5 μm.

Expression of pSipA-phiLOV was induced using IPTG, and fluorescence levels were measured to demonstrate successful expression and secretion of the phiLOV-tagged SipA by *S*. Typhimurium ΔSipA/pSipA-phiLOV (Fig. 3A). Increased fluorescence was evident in ΔSipA/pSipA-phiLOV bacterial cultures compared to the ΔSipA culture, and notably, this increase was particularly pronounced in the supernatant, suggesting that the bacteria were successfully secreting the tagged effector. Polar foci of SipA-phiLOV were also visualized within bacteria by microscopy, and we speculate that these are likely to be accumulations of SipA-phiLOV at the T3SS presecretion (Fig. 3B). These foci were absent in the ΔSipA/pT7-phiLOV and VV341/pSipA-phiLOV strains, which lack SipA and a functional T3SS, respectively.

**FIG 4:**
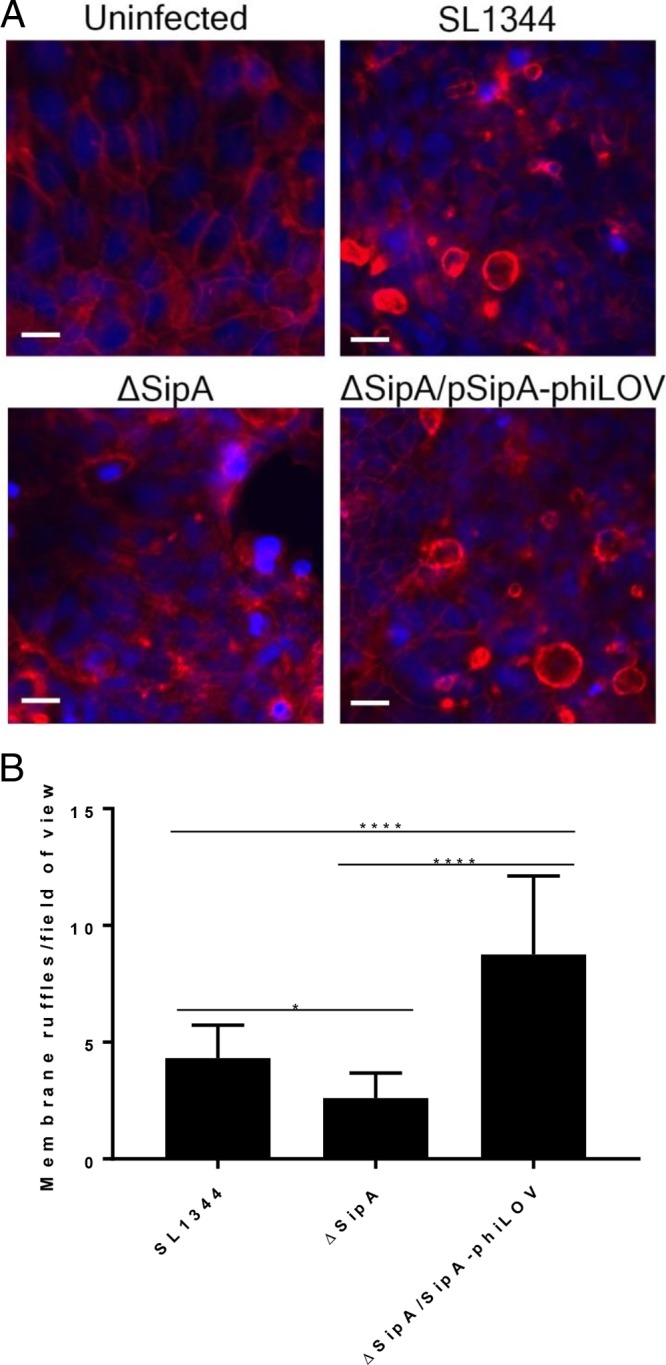
SipA-phiLOV induces membrane ruffling in T84 intestinal epithelial cells. (A) SipA-phiLOV, in a similar manner to that for SipA from wild-type SL1344, was seen to induce membrane ruffles after translocation into T84 epithelial cells. These ruffles were absent in the uninfected monolayers and were seen at lower frequency in the ΔSipA monolayers. Scale bar, 20 μm. (B) Distinct membrane ruffles per field of view were counted, and the average number of ruffles per 25 fields of view is depicted. Statistically significant differences are denoted. *P* values: *, <0.05; **, <0.01; ***, <0.001; ****, <0.0001.

Membrane ruffling is a well-characterized function of the actin binding C terminus of SipA. Thus, to demonstrate that SipA-phiLOV retained this function despite the presence of a C-terminally located phi-LOV tag, we assayed its ability to induce membrane ruffling in intestinal epithelial cells (5, 26). A blinded analysis of images from infected cells revealed that the number of membrane ruffles was reduced in the ΔSipA strain compared to SL1344 (Fig. 4A and B). As expected, the ΔSipA/pSipA-phiLOV strain rescues this defect and in fact induced a significantly greater number of membrane ruffles than even the wild-type strain, indicating that SipA-phiLOV was fully functional.

### Visualizing SipA-phiLOV *in vitro* and *ex vivo*.

**FIG 5:**
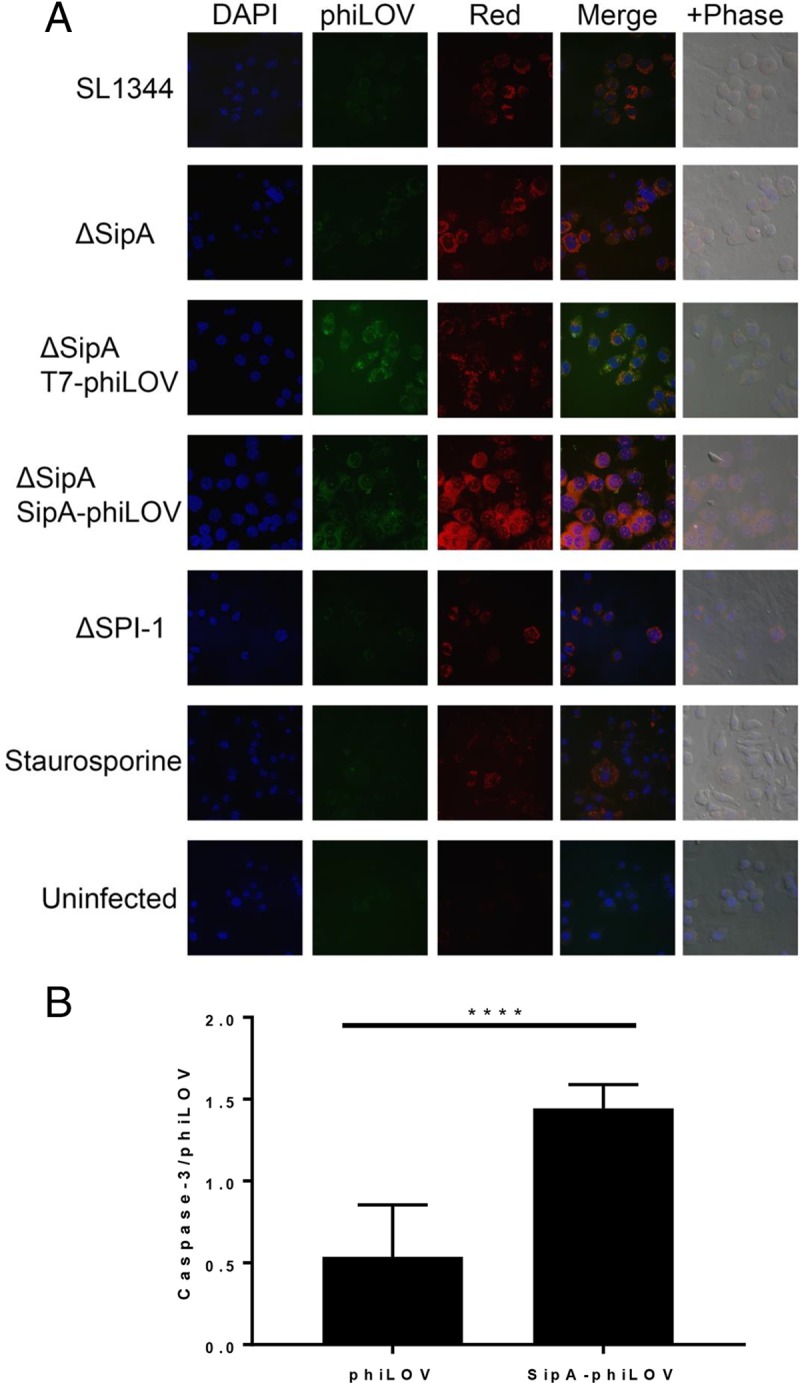
Expression of SipA-phiLOV in RAW264.7 macrophages results in increased levels of activated caspase-3. (A) Infected macrophages were fixed after staining for activated caspase-3 (red) at 2 hpi. High levels of phiLOV (green) expression could be seen in the ΔSipA strain complemented with pSipA-phiLOV and the control pT7-phiLOV plasmids. Nuclei are stained blue with the nuclear stain 4′,6′-diamidino-2-phenylindole (DAPI). (B) Twenty-five fields of view were taken from both phiLOV-infected samples, and fluorescent signals were quantified. To ensure comparability between samples, only fields of view with between 15 and 30 cells were used for analysis. Despite there being on average 1.8-fold more phiLOV signal in the ΔSipA/pT7-phiLOV-infected cells, ΔSipA/pSipA-phiLOV induced 1.6-fold more activated caspase-3. Statistically significant differences detected by Student's *t* test are denoted. *P* values: *, <0.05; **, <0.01; ***, <0.001, ****, <0.0001.

Having established that the ΔSipA/pSipA-phiLOV strain was functioning as expected, we went on to assess the role of SipA in caspase-3 induction *in vitro* in RAW264.7 cells and *ex vivo* in an ileal loop model of infection. To facilitate *in vitro* visualization of SipA colocalization with activated caspase-3, the ΔSipA/pSipA-phiLOV strain was used to infect RAW264.7 macrophages, and imaging was carried out on cells fixed at 2 hpi. Caspase-3 activation was visualized through addition of a fluorescent Image-IT Live imaging substrate (Molecular Probes). Activation of caspase-3 was observed in all infected cells, as expected, but was absent in uninfected cells (Fig. 5A). In ΔSipA/pSipA-phiLOV-infected cells, the phiLOV signal was visible alongside active caspase-3, and this was quantified to show a significant increase in caspase-3 relative to phiLOV signal in these cells compared to a the control phiLOV-secreting ΔSipA/pT7-phiLOV strain (Fig. 5B). This clearly demonstrated that SipA was inducing caspase-3 activation in macrophages.

**FIG 6:**
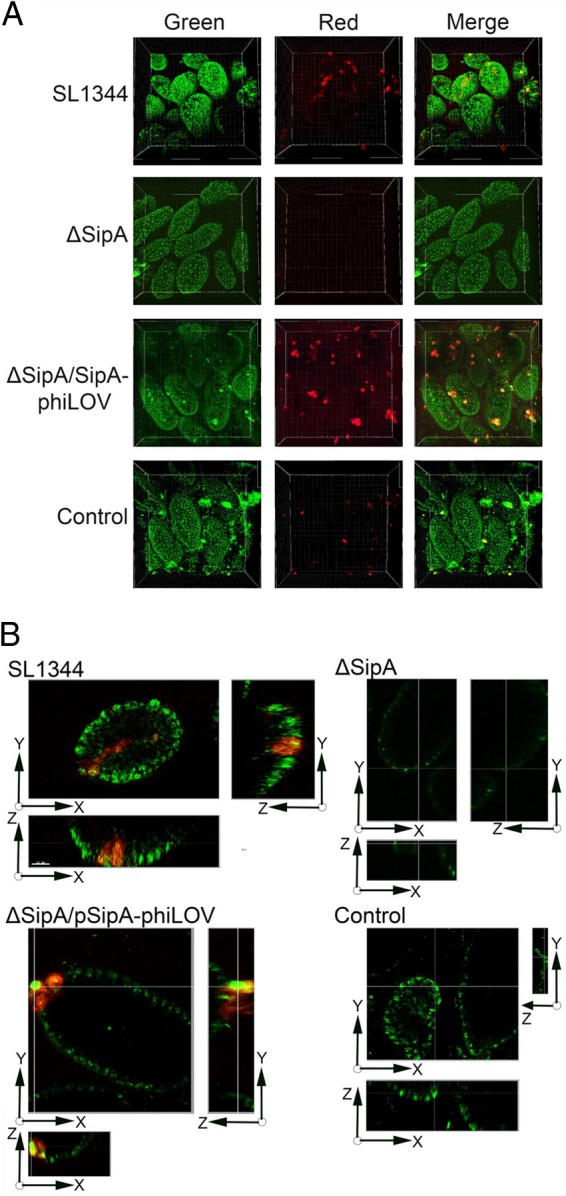
Multiphoton laser scanning microscopy (MPLSM) of SipA-phiLOV induction of caspase-3 activation *ex vivo*. (A) The SL1344, ΔSipA, and ΔSipA/SipA-phiLOV strains were used to infect an ileal loop model, and caspase-3 activation was detected with a laser scanning multiphoton microscope. Active caspase-3 was detected in epithelial cells at the tips of numerous villi in both SL1344- and ΔSipA/SipA-phiLOV-infected loops but was absent in ΔSipA and control loops. In ΔSipA/SipA-phiLOV-infected loops this signal colocalized with a strong green fluorescence, visible over native tissue fluorescence, and this was not seen in SL1344 infection (see Fig. S2 in the supplemental material for additional images). To determine the localization of food boluses observed in the control intestine and distinguish their autofluorescence from that attributable to phiLOV or active caspase-3, 3D videos to show their intraluminal location were prepared. Corresponding videos for each infection were also made (see Movies S1 to S4 in the supplemental material). (B) Using the Section View mode in Imaris software, distinct points within villi were inspected by showing the cut-through point in the *x*, *y*, and *z* axes simultaneously. Representative images are shown, demonstrating the strong intracellular signal attributable to SipA-phiLOV. This signal is absent in SL1344 villi where caspase-3 is present, and no visible active caspase-3 is present in ΔSipA-infected or control villi.

In order to gain a better insight into caspase-3 activation and the role it plays in infection in the intestine, SipA-phiLOV induction of caspase-3 was imaged in the murine intestine through MPLSM. Ileal loops were prepared *ex vivo* and infected with the SL1344, ΔSipA, or ΔSipA/SipA-phiLOV strain in the presence of the fluorescent Image-IT caspase-3 imaging substrate (Life Technologies). An uninfected loop was used as a control. Activation of caspase-3 was noted in epithelial cells within the loops after 30 min of infection, but in the ΔSipA/SipA-phiLOV-infected loops this activation was accompanied by an intense green fluorescent signal within caspase-3-positive cells that was absent in SL1344 loops (Fig. 6A). This signal was also absent in ΔSipA-infected loops, which additionally lacked the caspase-3-positive cells visible in the other infected loops. Due to the high background autofluorescence characteristic of MPLSM (and which has been reduced in Fig. S2 in the supplemental material to aid visualization), further three-dimensional (3D) rendering of images was carried out to confirm colocalization of the intense green fluorescent staining seen in ΔSipA/SipA-phiLOV-infected sections with active caspase-3 (Fig. 6B). This allowed viewing of infected loops in the *xy*, *xz*, and *yz* planes and demonstrated that the strong green fluorescent signal was emanating from caspase-3-positive cells in ΔSipA/SipA-phiLOV- but not SL1344- or ΔSipA-infected loops. Intense green/red fluorescent signal was observed in the uninfected control intestinal loops, but these pockets of fluorescence were established to be boluses of food remaining in the uninfected intestine. This was further confirmed by 3D rendition of the intestinal sections into short movies allowing rotation of the sections, which established the signal seen in uninfected samples was free in the intestinal lumen, unlike that seen in the infected samples (see Movies S1 to S4 in the supplemental material).

Activation of caspase-3 in both SL1344- and ΔSipA/SipA-phiLOV-infected loops occurred in concentrated focal points, often at the tips of villi where a number of cells in close proximity were affected. These infected areas may be linked to the “bystander effect” of caspase-3, where nearby cells are induced to undergo apoptosis through an as-yet-unknown signal from an apoptotic cell (27, 28). These areas of apoptotic cells may provide weak spots where *S*. Typhimurium can more easily transverse the intestinal barrier.

## DISCUSSION

SipA, the first effector delivered into host cells during *S*. Typhimurium infection, represents a crucial focal point in mediating the balance between establishment of a successful infection or the removal of bacteria. While the roles of effector proteins from *S*. Typhimurium have been described in detail, the finding that these effectors are multifunctional with individual functional domains has added an exciting layer of intrigue to their potential functions (10, 29). SipA has an established role as an actin-polymerizing protein and more recently as an inducer of inflammation with regards its role in the intestinal epithelium (5, 11). Although its perinuclear positioning in HeLa cells and fibroblasts has been noted, there is no evidence of a direct role for SipA in cells outside the intestinal epithelium (7–9, 16, 17).

During infection, SipA was seen to have a pronounced effect on caspase-3 activation in macrophages, with a near doubling of caspase-3 activation in response to SipA over the first 2 h of infection. This mirrored the temporal effect of SipA on caspase-3 seen over the first 5 h in intestinal epithelial cells (10). While temporal dependence of caspase-3 activation on SipA was noted at this lower, and probably more physiologically relevant, MOI, it disappeared at a higher MOI. The absence of the effect at higher MOI was most likely due to stress induced by the increased metabolic burden that a heavy infective dose places on infected macrophages, with programmed cell death (PCD) masking any subtle effects induced by effectors such as SipA (30–32).

The initial SipA-dependent spike in caspase-3 activity could in theory benefit either the host or pathogen, as caspase-3 plays multiple roles within host cells beyond apoptosis and induction of its activity during infection affects cell proliferation, differentiation, immunomodulation, macrophage migration, and signal transduction (33, 34). Therefore, caspase-3 activation by SipA likely has profound effects on the immune response early in infection. Additionally, host cells have adapted to detect bacterial invasion attempts using a T3SS, inducing oncosis in response to T3SS insertion and thus preventing bacterial entry and growth within the targeted host cell (35). SipA, as the first effector delivered through the T3SS, may play a similar role as an early detectable signal to the host of bacterial invasion, inducing apoptosis in the targeted cell as a host anti-invasion response.

Caspase-3 may also, as we previously demonstrated in the intestinal epithelium, inadvertently promote infection through processing bacterial effector proteins as they are translocated into host cells (10). However, in macrophages this seems counterintuitive given that processing of SipA would release the C-terminal actin binding domain, inducing actin polymerization, which in macrophages is a key innate immune mechanism for controlling Salmonella infection (36). Alternatively, as caspase-3 activity is tightly regulated, requiring a threshold level to initiate apoptosis in a host cell, slight alterations in its activity can have dramatic effects. SipA in increasing concentrations may move the level of caspase-3 activity beyond a threshold level and toward completion of apoptosis. With levels of SipA directly related to the number of bacteria within a cell, this would prevent numerous bacteria occupying the same cells simultaneously, thus disseminating the infective load and increasing the potential for success of the infection.

The extrabacterial release of up to 90% of SPI-1 effectors during infection remains a surprising and unexplained phenomenon, as rather than there being tight cooperative secretion/translocation control of effector expression, it appears to be indiscriminate (3). Other bacterial pathogens such as Fusobacterium and Streptococcus spp., release effectors that directly target PMNs, inducing apoptosis or necrosis after their binding or uptake by PMNs (18). This strategy maintains PMNs at a distance from where damage to bacteria through the release of toxic enzymes or neutrophil extracellular traps is limited (37). However, during *S*. Typhimurium infection, no increase in PCD was observed whether or not effectors, or indeed whole bacteria, were present with PMNs in culture (Fig. 3). Therefore, in this study at least, extrabacterial effectors appear to play no role in the intestine in regard to *S*. Typhimurium protection, unlike the case for other pathogens.

In order to visualize the process of SipA induction of caspase-3 during infection by *S*. Typhimurium, the novel phiLOV fluorescent tag was added to the C terminus of the protein (19). This tag, the first fluorescent tag known to be delivered through a T3SS during infection due to its small size, can function independently of oxygen, meaning that it is an excellent tag for imaging infection (20, 25). Additionally it avoids many of the drawbacks associated with other means of imaging infection, with the tag not dependent on activation or binding to other proteins for induction of its fluorescence upon entry into the target host cell (24, 38). SipA-phiLOV accumulated at focal points at the poles of expressing bacteria, and these foci were absent in strains that expressed phiLOV without SipA or which lacked a functional T3SS. We speculate that these polar foci are beneath the T3SS in anticipation of secretion, as has been reported in Shigella flexneri for IpaC and for other secretion systems (39–41). This polar localization of IpaC in S. flexneri was independent of any protein aggregation due to expression of tagged IpaC from a plasmid, although in S. flexneri the majority of bacteria expressed IpaC at a single pole. SipA-phiLOV functionality and secretion were established through membrane ruffling assays in intestinal epithelial cells, indicating that phiLOV was not interfering with the actin binding role of SipA, which is dependent on the C-terminal structure. Without a functional SipA or SipA-phiLOV, the membrane ruffles induced in intestinal epithelial cells during invasion were fewer, were of random size, and often appeared to be of irregular shape (26). The residual membrane ruffling in the ΔSipA mutant is likely due to effectors such as SipC which also polymerize actin (42).

Spinning-disc confocal microscopy allowed visualization of caspase-3 enzyme activity in infected macrophages (Fig. 5A). As previously shown (Fig. 1), the presence of SipA induced a significant increase in caspase-3 activity which could be visualized in both wild-type-infected macrophages and those infected with the ΔSipA/SipA-phiLOV strain (Fig. 5). As expected, caspase-3, which is constitutively active in mammalian cells, could still be visualized to some extent in all infected or treated cells and even in uninfected cells. To definitively show that the effect was SipA dependent and was independent of the added phiLOV tag, the level of phiLOV fluorescence was directly correlated with that of caspase-3 activity (Fig. 5B). This indicated that there was more than 2.5-fold more caspase-3 activity relative to phiLOV signal in cells where SipA was present. This indicated that the phiLOV tag offered an enhanced imaging capability over other techniques such as GFP tagging for Salmonella infection, as it could be delivered attached to an effector protein during infection and not interfere with the role of the effector during infection.

To image *ex vivo* intestinal loops, multiphoton laser scanning microscopy (MPLSM) was used, as although the spectral properties of phiLOV overlap those of GFP, its emission spectrum is at 528 nm as opposed to 508 nm for GFP. MPLSM therefore offered the advantage that the laser could be tuned to different wavelengths, allowing selection of the optimal wavelength for phiLOV, and imaging could be carried out at significant depths into tissue, up to 1,000 μm. MPLSM has previously been used to successfully image both bacterial infection and the host response *ex vivo* or *in vivo* (43–45). However, a single effector has not previously been studied through MPLSM, as previous studies have relied on bacteria expressing chromosomal GFP. Caspase-3 activity was visible in both SL1344- and ΔSipA/pSipA-phiLOV-infected loops but not the ΔSipA-infected or uninfected samples (Fig. 6). A strong fluorescent signal from SipA-phiLOV could be seen colocalizing in infected villus cells with activated caspase-3, and this was confirmed by 3D rendering of images, reduction of background autofluorescence, and the use of 3D movies to establish the intracellular nature of this signal and its absence in the other infected or untreated samples (Fig. 6; see Fig. S2 and Movies S1 to S4 in the supplemental material). This signal could be seen across a number of villi, and these infected cells often appeared in clusters which would lead to significant breaches in the intestinal barrier function. Additionally, activated caspase-3 released into the intestine from these infected or dying cells can cleave tight-junction proteins and allow migration of *S*. Typhimurium through the intestinal barrier (46, 47). These key developmental MPLSM data indicate that the phiLOV tag and related flavin-based fluorescent reporters will be novel tools for use in future *in vivo* studies, potentially allowing tracking of cells where effectors are activating specific host proteins or pathways.

This work has established a crucial role for SipA in establishing infection by *S*. Typhimurium in the intestine through mediating the activation of caspase-3 in distinct cell types. This effect is dependent on individual cell types, with macrophages and intestinal epithelial cells undergoing increased caspase-3 activation whereas PMNs remain unaffected. Furthermore, we have imaged these interactions for the first time using the phiLOV tag attached to SipA and have showed that this tag shows great promise for tagging and tracking bacterial proteins during infection in both *in vitro* and *in vivo*.

## MATERIALS AND METHODS

### Bacterial strains and growth conditions.

Wild-type *S*. Typhimurium SL1344 and mutants thereof were used throughout this study. All cloning was carried out in E. coli strain BL21(DE3) (Life Technologies). Strains for infection were grown for 12 h at 37°C in a shaking incubator at 180 rpm in phenol red-free RPMI 1640 medium (Sigma) supplemented with 2 mM l-glutamine and 3% fetal calf serum (FCS). Seed cultures were then back-diluted 1:10 and grown overnight at 37°C in 10-ml stationary cultures.

### Plasmid construction.

SipA was amplified using primers T7SipApLOVFor (5′-GGTGGGAGCTCATGGTTACAAGTGTAAGGACTCAGCC-3′) and T7SipApLOVRev (5′-GGTGGCTCGAGACGCTGCATGTGCAAGCCATCAACGGTAGTAATAA-3′) bearing SacI and XhoI sites, respectively. The amplified fragment was cloned using the SacI and XhoI sites into the vector pT7-phiLOV (GenScript), which bears an ampicillin resistance cassette. This plasmid was designed to add a C-terminal phiLOV tag to cloned effector proteins with expression driven by the IPTG-inducible T7 promoter.

### Bacterial growth conditions.

Plasmid-bearing E. coli strains were grown with aeration in Luria-Bertani (LB) broth containing ampicillin (50 μg/ml). For infection the pT7-SipA-phiLOV plasmid (now termed pSipA-phiLOV) was cloned into the ΔSipA *S*. Typhimurium strain EE633, where it was selected for again in LB and ampicillin (50 μg/ml). For infection with plasmid-bearing strains, bacteria were grown as described above but with ampicillin, with protein expression induced by the addition of 1 mM IPTG at 3 h prior to infection.

### Characterization of phiLOV expression.

Expression of phiLOV and its extracellular release were tracked over time through assaying for increases in fluorescence intensity both in whole cells and in culture supernatants as previously described (20). Aliquots of culture were taken at various time points, and to assay supernatants, cultures were centrifuged at 14,000 × *g* for 5 min to remove all bacteria before assaying. Assays were carried out using a FluoStar Optima microplate reader (BMG Labtech).

### Maintenance of cell lines and infection.

RAW264.7 macrophages obtained from the European Collection of Cell Cultures (ECACC) were maintained in RPMI 1640 (Sigma) phenol red-free medium supplemented with 10% FCS, 2 mM l-glutamine, and penicillin (50 units/ml) and streptomycin (50 μg/ml) (both from Sigma). The T84 human intestinal epithelial cell line obtained from the American Type Culture Collection (ATCC) was maintained in a 50:50 mixture of Dulbecco's modified Eagle's medium and Ham's F-12 medium (both from Sigma) supplemented with 10% FCS, 2 mM l-glutamine, 15 mM HEPES buffer (pH 7.5), 14 mM sodium bicarbonate, and penicillin (50 units/ml) and streptomycin (50 μg/ml) (Sigma). Cell lines were maintained at 37°C and 5% CO_2_ with regular medium changes. RAW264.7 cells were passaged before reaching 90% confluence, while T84 cells were passaged at 70% confluence. RAW264.7 cells were seeded at 1 × 10^6^ cells per well in glass-bottom dishes (WillCo Wells BV) for imaging or at 1 × 10^5^ cells per well on glass coverslips in a 24-well plate 48 h prior to infection. At 24 h prior to infection, RAW264.7 cells were treated with 100 ng/ml lipopolysaccharide to induce activation. T84 epithelial cells were seeded at 1 × 10^6^ cells per glass-bottom dish or at 1 × 10^5^ per well on glass coverslips 7 days prior to infection. The medium was changed at days 1 and 4 and a 100% confluent polarized monolayer of cells allowed form. Infections were carried out at multiplicities of infection (MOIs) of 10 or 100 for RAW264.7 cells or 100 for intestinal epithelial cells. After 1 h, the bacteria that had not been internalized were killed by adding 50 ng/ml gentamicin sulfate (Sigma-Aldrich), and the infection was allowed to proceed.

Human blood was obtained from the Glasgow Blood Transfusion Services. PMNs were extracted as previously described and seeded in 24-well tissue culture plates in phenol red-free RPMI 1640 supplemented with 3% FCS and 2 mM l-glutamate (48). PMNs were then treated with either whole bacteria or effector proteins isolated from the supernatant of the same cultures. For whole bacteria, an MOI of 100 was used. For purified *S*. Typhimurium effectors, 5 ml of supernatant was cleared of bacteria through centrifugation at 14,000 × *g* for 5 min. Supernatants were passed through a 0.2-μm-pore filter to remove any remaining bacteria, and the protein content was calculated by Bradford assay. Protein levels across all samples were equilibrated to 1 mg/ml and 200 μl of these suspensions added to the appropriate wells. Supernatants and lysates were collected and prepared as before with cells lysed in phosphate-buffered saline (PBS) supplemented with 2% Triton (49).

### Staining of cellular proteins.

Caspase-3 was stained using the Image-iT Live Red caspase-3 and -7 detection kit (Life Technologies) for both fixed- and live-cell imaging. Staining was carried out according to the manufacturer's protocol. Actin was stained using rhodamine phalloidin according to the manufacturer's protocol (Life Technologies).

### Imaging of *S*. Typhimurium infection.

Membrane ruffles on T84 cells were imaged using a DeltaVision RT live-imaging system (Applied Precision) with WeatherStation environmental control. RAW264.7 cells fixed in 2% paraformaldehyde were imaged using a spinning-disc confocal microscope (microscope, inverted, Zeiss Axio Observer.Z1; spinning disc unit, Yokogawa CSU-X1A 5000; camera, Photometrics Evolve 512 Delta EMCCD). Z-stacking was employed where necessary, and images were analyzed using both Imaris (Bitplane) and Fiji software packages.

### Multiphoton laser scanning microscopy to detect SipA-phiLOV.

Eight-week-old female C57BL/6 mice were euthanized by cervical dislocation, and sections of colon were ligated and then immediately removed and maintained in warmed Hanks balanced salt solution (HBSS) (Gibco). Approximately 5 × 10^7^ SL1344, ΔSipA, or ΔSipA/pSipA-phiLOV cells were injected intraluminally along with Image-iT Live Red caspase-3/-7 substrate (Molecular Probes). Multiphoton imaging through the intestinal wall was carried out using a Zeiss LSM7 MP system equipped with a 20×/1.0 NA water immersion objective lens (Zeiss UK, Cambridge, UK) and a tunable titanium-sapphire solid-state two-photon excitation source (Chameleon Ultra II; Coherent Laser Group, Glasgow, UK) and optical parametric oscillator (OPO) (Coherent Laser Group). Images were reconstructed postmicroscopy into 3D images using ZEN software (Zeiss).

### Enzyme activity assays after *S*. Typhimurium infection.

Cell lysates were prepared postinfection by removing supernatants and scraping off cells in 2% Triton in PBS at pH 7.5. Both supernatants and lysates were stored at −20°C until needed. Lactate dehydrogenase (LDH) activity as a measure of cytoxicity was measured in cell supernatants according the manufacturer's protocol (LDH cytotoxicity assay kit; Abcam). Caspase-3 activity was measured using the Apo-One homogenous caspase-3 activity kit (Promega). Postmeasurement caspase-3 activity was corrected for protein concentration (bicinchoninic acid [BCA] protein assay kit; Pierce) and expressed as caspase-3 activity relative fluorescent units per milligram of protein. All enzyme activities, both fluorescent and absorbent, were measured using a FluoStar Optima microplate reader (BMG Labtech).

### Ethics statement.

Approval for these procedures was given prior to their initiation by an internal University of Glasgow ethical review board. All procedures involving animals were carried out in accordance with the relevant guidelines and regulations as outlined by the U.K. Home Office under PPL 7008584.

### Statistical analysis.

All data presented are representative of at least three independent experiments, expressed as the mean value ± standard deviation (SD), and analyzed using an analysis of variance (ANOVA) multiple-comparison test in GraphPad Prism software unless otherwise stated. *P* values less than 0.05 were considered significant.

## Supplementary Material

Supplemental material
