# Supplementary material for: SipA Activation of Caspase-3 Is a Decisive Mediator of Host Cell Survival at Early Stages of Salmonella enterica Serovar Typhimurium Infection
Source: Infect Immun. 2017 Aug 18;85(9):e00393-17. doi: 10.1128/IAI.00393-17 (PMC5563584; doi:10.1128/IAI.00393-17)
Supplement: Supplemental material [file supp_85_9_e00393-17__index.html]

SipA Activation of Caspase-3 Is a Decisive Mediator of Host Cell Survival at Early Stages of Salmonella enterica Serovar Typhimurium Infection — Supplemental material 

# SipA Activation of Caspase-3 Is a Decisive Mediator of Host Cell Survival at Early Stages of Salmonella enterica Serovar Typhimurium Infection

## Supplemental material

- Supplemental file 1 -

  Fig. S1. Recovery of bacteria from infected macrophages after infection. Fig. S2. Multiphoton laser scanning microscopy of *S.* Typhimurium-infected ileal loops *ex vivo*. Legends for Movies S1 to S4.

  PDF, 247K
- Supplemental file 2 -

  Movie S1. Multiphoton laser scanning microscopy of a Δ*sipA* strain-infected ileal loop *ex vivo*.

  AVI, 12M
- Supplemental file 3 -

  Movie S2. Multiphoton laser scanning microscopy of a Δ*sipA*/pSipA-phiLOV strain-infected ileal loop *ex vivo*.

  AVI, 12M
- Supplemental file 4 -

  Movie S3. Multiphoton laser scanning microscopy of an SL1344-infected ileal loop *ex vivo*.

  AVI, 19M
- Supplemental file 5 -

  Movie S4. Multiphoton laser scanning microscopy of an uninfected ileal loop *ex vivo*.

  AVI, 17M
