# Supplementary material for: SipA Activation of Caspase-3 Is a Decisive Mediator of Host Cell Survival at Early Stages of Salmonella enterica Serovar Typhimurium Infection
Source: Infect Immun. 2017 Aug 18;85(9):e00393-17. doi: 10.1128/IAI.00393-17 (PMC5563584; doi:10.1128/IAI.00393-17)
Supplement: Supplemental material [file IAI.00393-17_zii999092114s1.pdf]

Supplementary Figures

Figure S1

A

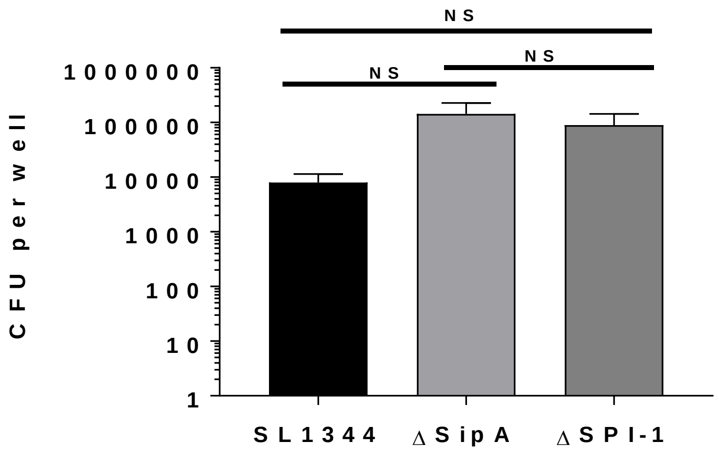

B

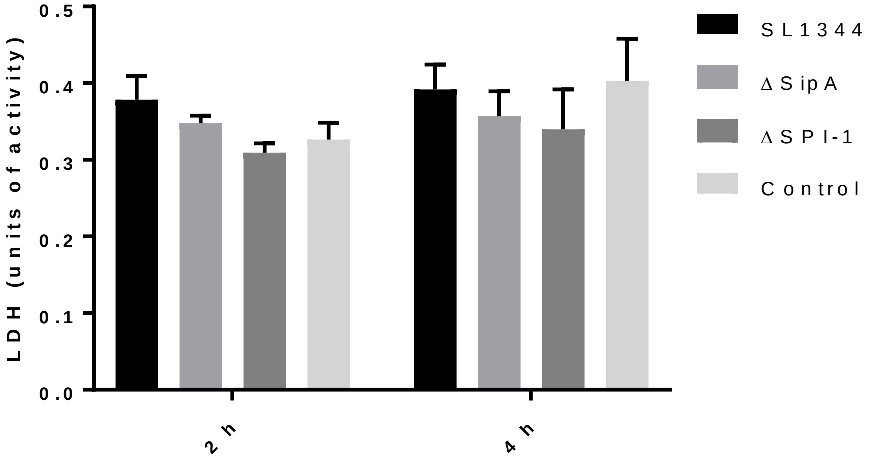

Figure S1: Recovery of bacteria from infected macrophages after infection. (A) Macrophages were infected at an MOI of 10 and infection allowed proceed for 1 h. Gentamycin was added and 0.5 h later intracellular bacteria were counted. Data is representative of three separate experiments. Although a trend towards higher numbers of intracellular bacteria lacking SipA in RAW264.7 cells was noted it was not significant. (B) The SipA induced apoptotic effect in RAW264.7 macrophages is independent of necrosis.

Quantification of LDH release from RAW264.7 macrophages post-infection at an MOI of 10 showed that there was no statistically significant difference between wild type SL1344 and ΔSipA infected macrophages.

Figure S2

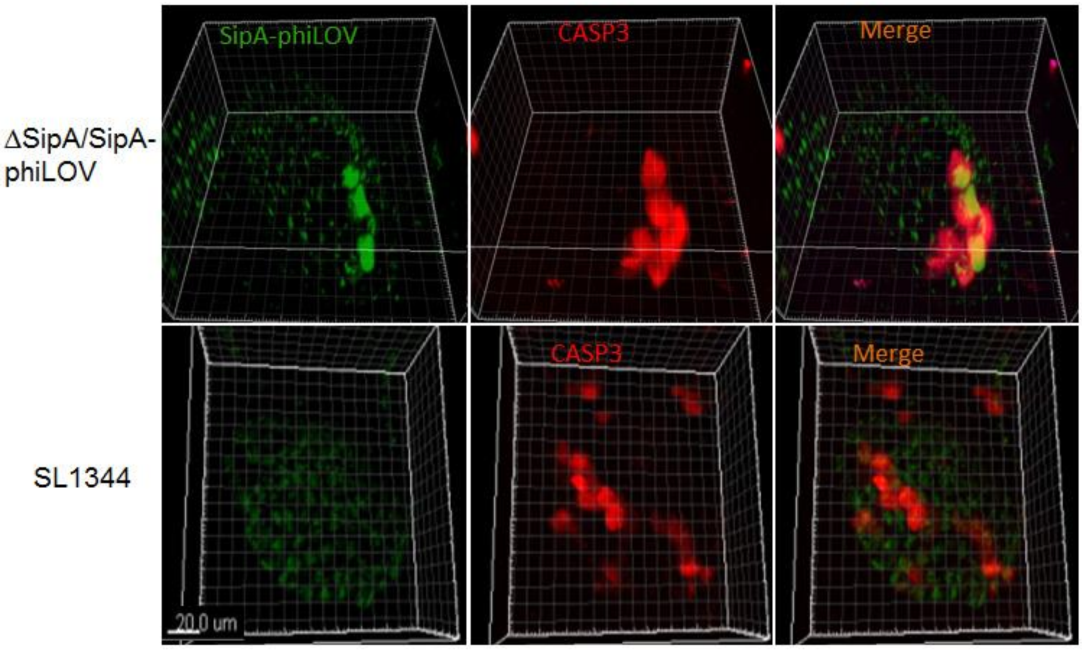

Figure S2: Multiphoton laser scanning microscopy of S. Typhimurium infected ileal loops *ex vivo*. Caspase-3 activation was noted in intestinal epithelial cells infected with both SL1344 and ΔSipA/pSipA-phiLOV but not ΔSipA infected or control loops (Fig. 6). The influence of background auto fluorescence and second harmonic generation due to laser excitation of tissues were reduced by further post-imaging analysis using Imaris imaging software allowing the visualisation of villus structure and better imaging of the phiLOV signal and its co-localization with active caspase-3.

Supplemental Figure Legends

Movie S1: Multiphoton laser scanning microscopy of a ΔSipA infected ileal loop *ex vivo*. Post imaging a 3D movie was constructed using Imaris imaging software. Background auto fluorescence allows visualisation of the structure of numerous villi but caspase-3 activity is not visible.

Movie S2: Multiphoton laser scanning microscopy of a ΔSipA/pSipA-phiLOV infected ileal loop *ex vivo*. Post imaging a 3D movie was constructed using Imaris imaging software. Background auto fluorescence allows visualisation of the structure of villi whilst caspase-3 activity (red) is visible in distinct intestinal cells and co-localizes with intense green fluorescence (phiLOV). Pockets of caspase-3 positive cells are visible with, in some cases, neighbouring caspase-3 positive cells not exhibiting a phiLOV signal.

Movie S3: Multiphoton laser scanning microscopy of an SL1344 infected ileal loop *ex vivo*. Post imaging a 3D movie was constructed using Imaris imaging software. Background auto fluorescence allows visualisation of the structure of villi whilst caspase-3 activity (red) is visible, but notably without the intense green fluorescence noted in Movie S2 with phiLOV, which is absent from all caspase-3 positive cells.

Movie S4: Multiphoton laser scanning microscopy of an uninfected ileal loop *ex vivo*. Post imaging a 3D movie was constructed using Imaris imaging software. Background auto fluorescence allows visualisation of the structure of villi but both caspase-3 activity and phiLOV signal are notably absent. Food boluses can be seen in the intestine and whilst these are exhibiting some limited auto fluorescence it can be seen that unlike the signals in Movie S2 this signal is free floating in the intestine.
